# Supplementary material for: Profiles of Cytokines Secreted by ARPE-19 Cells Exposed to Light and Incubated with Anti-VEGF Antibody
Source: Biomedicines. 2021 Sep 27;9(10):1333. doi: 10.3390/biomedicines9101333 (PMC8533158; doi:10.3390/biomedicines9101333)
Supplement: Supplementary file 1 [file biomedicines-09-01333-s001.zip › biomedicines-1373529-supplementary.pdf]

Supplementary Table S1. Comparisons of cytokine levels in culture supernatant of ARPE-19 cells incubated with aflibercept at various concentrations in the dark

A

4 groups	*p* value
IL-6	0.060
IL-8	0.051
**IL-12**	**2.05×10^{-6}**
IL-17A	0.196
bFGF	0.119
MCP-1	0.418
**VEGF**	**4.13×10^{-5}**

B

IL-12	7.81 μg-A	31.3 μg-A	0.125 mg-A	0.50 mg-A
**7.81 μg-A**	—	**3.87×10^{-7}**	**3.87×10^{-7}**	**3.87×10^{-7}**
**31.3 μg-A**	**	—	1.000	1.000
**0.125 mg-A**	**		—	1.000
**0.50 mg-A**	**			—

C

VEGF	7.81 μg-A	31.3 μg-A	0.125 mg-A	0.50 mg-A
**7.81 μg-A**	—	**1.03×10^{-4}**	**8.61×10^{-5}**	**8.12×10^{-5}**
**31.3 μg-A**	**	—	0.571	0.381
**0.125 mg-A**	**		—	0.967
**0.50 mg-A**	**			—

ARPE-19 cells were cultured with 7.81 μg/mL, 31.3 μg/mL, 0.125 mg/mL or 0.50 mg/mL of aflibercept in the dark. Four groups consisted of aflibercept-exposed cultures at indicated concentrations. (**A**) cytokine levels among the 4 groups were compared by non-repeated measures ANOVA. (**B** and **C**) *P* values in all pairwise comparisons of IL-12 and VEGF-A levels among aflibercept-exposed cultures were obtained by Tukey-Kramer test. $n = 3$ in each group. A: aflibercept, mg: mg/mL, μg: μg/mL.

Supplementary Table S2. Comparisons of cytokine levels in culture supernatant of ARPE-19 cells incubated with aflibercept at various concentrations under light irradiation

A

4 groups	*p* value
**IL-6**	**0.015**
**IL-8**	**0.040**
IL-12	0.479
IL-17A	0.734
bFGF	0.154
**MCP-1**	**0.019**
**VEGF**	**0.046**

B

IL-6	7.81 µg-A	31.3 µg-A	0.125 mg-A	0.50 mg-A
**7.81 µg-A**	—	**0.013**	0.163	**0.037**
**31.3 µg-A**	*	—	0.095	0.489
**0.125 mg-A**			—	0.412
**0.50 mg-A**	*			—

C

IL-8	7.81 µg-A	31.3 µg-A	0.125 mg-A	0.50 mg-A
**7.81 µg-A**	—	**0.042**	0.117	0.058
**31.3 µg-A**	*	—	0.650	0.974
**0.125 mg-A**			—	0.849
**0.50 mg-A**				—

D

MCP-1	7.81 µg-A	31.3 µg-A	0.125 mg-A	0.50 mg-A
**7.81 µg-A**	—	**0.035**	0.085	**0.018**
**31.3 µg-A**	*	—	0.693	0.774
**0.125 mg-A**			—	0.292
**0.50 mg-A**	*			—

E

VEGF	7.81 µg-A	31.3 µg-A	0.125 mg-A	0.50 mg-A
**7.81 µg-A**	—	0.148	**4.97×10^{-2}**	0.061
**31.3 µg-A**		—	0.625	0.752
**0.125 mg-A**	*		—	0.993
**0.50 mg-A**				—

ARPE-19 cells were cultured with 7.81 μg/mL, 31.3 μg/mL, 0.125 mg/mL or 0.50 mg/mL of aflibercept under light irradiation. Four groups were composed of aflibercept-exposed cultures at indicated concentrations. (**A**) Cytokine levels among the 4 groups were compared. (**B** to **E**) All pairwise comparisons of IL-6, IL-8, MCP-1 and VEGF-A levels among aflibercept-exposed cultures were performed. $n = 3$ in each group.

Supplementary Table S3. Comparisons of cytokine levels in culture supernatant of ARPE-19 cells incubated with ranibizumab at various concentrations in the dark

A

4 groups	*p* value
**IL-6**	**0.002**
**IL-8**	**0.013**
**IL-12**	**1.08×10^{-4}**
IL-17A	0.239
**bFGF**	**0.021**
**MCP-1**	**0.005**
**VEGF**	**8.22×10^{-5}**

B

IL-6	1.95 μg-R	7.81 μg-R	31.3 μg-R	0.125 mg-R
1.95 μg-R	—	**0.006**	**0.007**	**0.001**
7.81 μg-R	**	—	0.990	0.076
31.3 μg-R	**		—	0.059
0.125 mg-R	**			—

C

IL-8	1.95 μg-R	7.81 μg-R	31.3 μg-R	0.125 mg-R
1.95 μg-R	—	0.702	0.143	**0.012**
7.81 μg-R		—	0.413	**0.025**
31.3 μg-R			—	0.097
0.125 mg-R	*	*		—

D

IL-12	1.95 μg-R	7.81 μg-R	31.3 μg-R	0.125 mg-R
1.95 μg-R	—	**0.021**	0.102	**2.71×10^{-4}**
7.81 μg-R	*	—	**0.004**	**1.14×10^{-4}**
31.3 μg-R		**	—	**0.001**
0.125 mg-R	**	**	**	—

E

bFGF	1.95 μg-R	7.81 μg-R	31.3 μg-R	0.125 mg-R
1.95 μg-R	—	0.128	0.084	**0.015**
7.81 μg-R		—	0.960	0.152
31.3 μg-R			—	0.240
0.125 mg-R	*			—

F

MCP-1	1.95 μg-R	7.81 μg-R	31.3 μg-R	0.125 mg-R
1.95 μg-R	—	**0.007**	**0.011**	**0.008**
7.81 μg-R	**	—	0.828	0.996
31.3 μg-R	*		—	0.914
0.125 mg-R	**			—

G

VEGF	1.95 μg-R	7.81 μg-R	31.3 μg-R	0.125 mg-R
1.95 μg-R	—	**3.51×10^{-4}**	0.050	**0.004**
7.81 μg-R	**	—	**0.001**	**8.59×10^{-5}**
31.3 μg-R		**	—	**0.001**
0.125 mg-R	**	**	**	—

ARPE-19 cells were cultured with 1.95 μg/mL, 7.81 μg/mL, 31.3 μg/mL or 0.125 mg/mL of ranibizumab in the dark. Four groups consisted of ranibizumab-exposed cultures at indicated concentrations. (**A**) Cytokine levels among the 4 groups were compared. (**B** to **G**) All pairwise comparisons of IL-6, IL-8, IL-12, bFGF, MCP-1 and VEGF-A levels among ranibizumab-exposed cultures were performed. *n* = 3 in each group. R: ranibizumab.

Supplementary Table S4. Comparisons of cytokine levels in culture supernatant of ARPE-19 cells incubated with ranibizumab at various concentrations under light irradiation

A

4 groups	*p* value
IL-6	0.490
IL-8	0.325
**IL-12**	**0.005**
IL-17A	0.415
bFGF	0.632
MCP-1	0.105
**VEGF**	**0.012**

B

IL-12	7.81 μg-A	31.3 μg-A	0.125 mg-A	0.50 mg-A
**7.81 μg-A**	—	0.739	**0.045**	**0.005**
**31.3 μg-A**		—	0.106	**0.009**
**0.125 mg-A**	*		—	0.080
**0.50 mg-A**	**	**		—

C

VEGF	7.81 μg-A	31.3 μg-A	0.125 mg-A	0.50 mg-A
**7.81 μg-A**	—	0.809	**0.047**	**0.014**
**31.3 μg-A**		—	0.099	**0.025**
**0.125 mg-A**	*		—	0.416
**0.50 mg-A**	*	*		—

ARPE-19 cells were cultured with 1.95 μg/mL, 7.81 μg/mL, 31.3 μg/mL or 0.125 mg/mL of ranibizumab under light irradiation. Four groups were composed of ranibizumab-exposed cultures at indicated concentrations. (**A**) Cytokine levels among the 4 groups were compared. (**B** and **C**) All pairwise comparisons of IL-12 and VEGF-A levels among ranibizumab-exposed cultures were performed. $n = 3$ in each group.
